# Ontogeny and social context regulate the circadian activity patterns of Lake Malawi cichlids

**DOI:** 10.1007/s00360-023-01523-3

**Published:** 2023-11-01

**Authors:** Evan Lloyd, Aakriti Rastogi, Niah Holtz, Ben Aaronson, R. Craig Albertson, Alex C. Keene

**Affiliations:** 1 Department of Biology, Texas A&M University, College Station, TX 77840 USA; 2 Organismic and Evolutionary Biology Graduate Program, University of Massachusetts, Amherst, MA 01003 USA; 3 Department of Biology, University of Massachusetts, Amherst, MA 01003 USA

**Keywords:** Circadian, Sleep, Evolution, Cichlid, Clock

## Abstract

Activity patterns tend to be highly stereotyped and critical for executing many different behaviors including foraging, social interactions, and predator avoidance. Differences in the circadian timing of locomotor activity and rest periods can facilitate habitat partitioning and the exploitation of novel niches. As a consequence, closely related species often display highly divergent activity patterns, suggesting that shifts from diurnal to nocturnal behavior, or vice versa, are critical for survival. In Africa’s Lake Malawi alone, there are over 500 species of cichlids, which inhabit diverse environments and exhibit extensive phenotypic variation. We have previously identified a substantial range in activity patterns across adult Lake Malawi cichlid species, from strongly diurnal to strongly nocturnal. In many species, including fishes, ecological pressures differ dramatically across life-history stages, raising the possibility that activity patterns may change over ontogeny. To determine if rest-activity patterns change across life stages, we compared the locomotor patterns of six Lake Malawi cichlid species*.* While total rest and activity did not change between early juvenile and adult stages, rest-activity patterns did, with juveniles displaying distinct activity rhythms that are more robust than adults. One distinct difference between juveniles and adults is the emergence of complex social behavior. To determine whether social context is required for activity rhythms, we next measured locomotor behavior in group-housed adult fish. We found that when normal social interactions were allowed, locomotor activity patterns were restored, supporting the notion that social interactions promote circadian regulation of activity in adult fish. These findings reveal a previously unidentified link between developmental stage and social interactions in the circadian timing of cichlid activity.

**Supplementary Information:**

The online version contains supplementary material available at 10.1007/s00360-023-01523-3.

## Introduction

Animals display remarkable diversity in rest-activity patterns (Keene and Duboue 2018; Joiner 2016). The timing of rest and activity can differ dramatically between closely related species, or even between populations of the same species, raising the possibility that it can be adaptive and subject to selection (Bhadra et al. 2017; Foulkes et al. 2016). Indeed, circadian regulation of locomotor activity is strongly associated with many factors critical in determining organismal fitness, including foraging strategy, social behavior, and predator avoidance (Kronfeld-Schor et al. 2017; Bass and Lazar 2016). Further, rest-activity patterns are acutely regulated by environmental factors and life-history traits that include food availability, social interactions, and age (Mohawk et al. 2012; Mattis and Sehgal 2016). Defining how complex environmental interactions regulate activity patterns is therefore critical to understanding behavioral adaptation and evolution.

There are over 35,000 teleost species, adapted to diverse habitats, and representing ~ 50% of vertebrate diversity (Froese et al. 2023). Many teleosts display robust diurnal locomotor rhythms including the goldfish (*Carassius Auratus*), river-dwelling populations of the Mexican tetra (*Astyanax mexicanus*), and the zebrafish (*Danio rerio*) (Sánchez-Vázquez et al. 1996; Duboué et al. 2011; Hurd et al. 1998; Zhdanova et al. 2001). Examples of nocturnal teleosts have also been identified including the plainfin midshipman, the Senegalese sole, and the doctor fish, *Tinca tinca* (Herrero et al. 2005). Other species, such as cavefish morphs of the Mexican tetra, *A. mexicanus,* and the Somalian cavefish, *Phreatichthys andruzzii,* and cave dwelling populations of *A. mexicanus*, have largely lost light-driven circadian regulation of behavior (Mack et al. 2021; Cavallari et al. 2011; Beale et al. 2013; Ceinos et al. 2018). Despite the characterization of species from disparate lineages/populations, a few studies have examined how developmental stage, or divergent ecological contexts, regulate sleep among closely related species. Understanding the contributions of phylogeny, ecology, and ontogeny variable rest-activity patterns represents a critical step in identifying conserved genetic and evolutionary features that may influence regulation of activity throughout vertebrates.

Lake Malawi cichlids exhibit unparalleled diversity in morphology and behavior among vertebrates (Salzburger 2018; Kornfield and Smith 2000; Kocher 2004). Observations at night suggest that adult cichlids may be diurnal as an evolved strategy for predator avoidance, at least for species occupying near-shore habitats (Fryer and Iles 1972). Specifically, Lake Malawi is home to endemic non-cichlid predators, including the Cornish jack *Mormyrops anguilloides,* which feeds in packs at night using weak electrical pulses thought to be undetectable by cichlids (Arnegard and Carlson 2005). We previously analyzed the activity patterns of 11 species of cichlids, from diverse habitats and distinct genetic lineages, and found significant variability ranging from highly nocturnal to diurnal, with many species exhibiting no differences in day or night activity (Lloyd et al. 2021). In a single identified nocturnal species, *Tropheops.* sp*.* “red cheek,’ the pattern held at two life-history stages (i.e., late juvenile vs. mature adult), and under different abiotic conditions (i.e., presence vs. absence of shelter) (Lloyd et al. 2021). However, for many species, there was no preference for light or dark activity, raising the possibility that diurnality and nocturnality are only present under specific environmental contents, or that they are absent in some species (Lloyd et al. 2021).

Here, we focused on the activity patterns of Lake Malawi cichlids, where many adult species seem to lack activity patterns during adulthood. All Lake Malawi cichlids are maternal mouth brooders, and we found that as newly emerged fry (i.e., early juvenile stage), all six species exhibited robust rest-activity patterns. Thus, we demonstrate ontogenic regulation of activity patterns across diverse cichlids. Furthermore, in a subset of species, we find that diurnal activity patterns of juveniles were largely restored is adult fish under social conditions. Together, these studies demonstrate the complexity and context dependency of the circadian regulation of rest activity in Lake Malawi cichlids.

## Results

### Total activity is similar between juvenile and adult cichlids

**Fig. 1:**
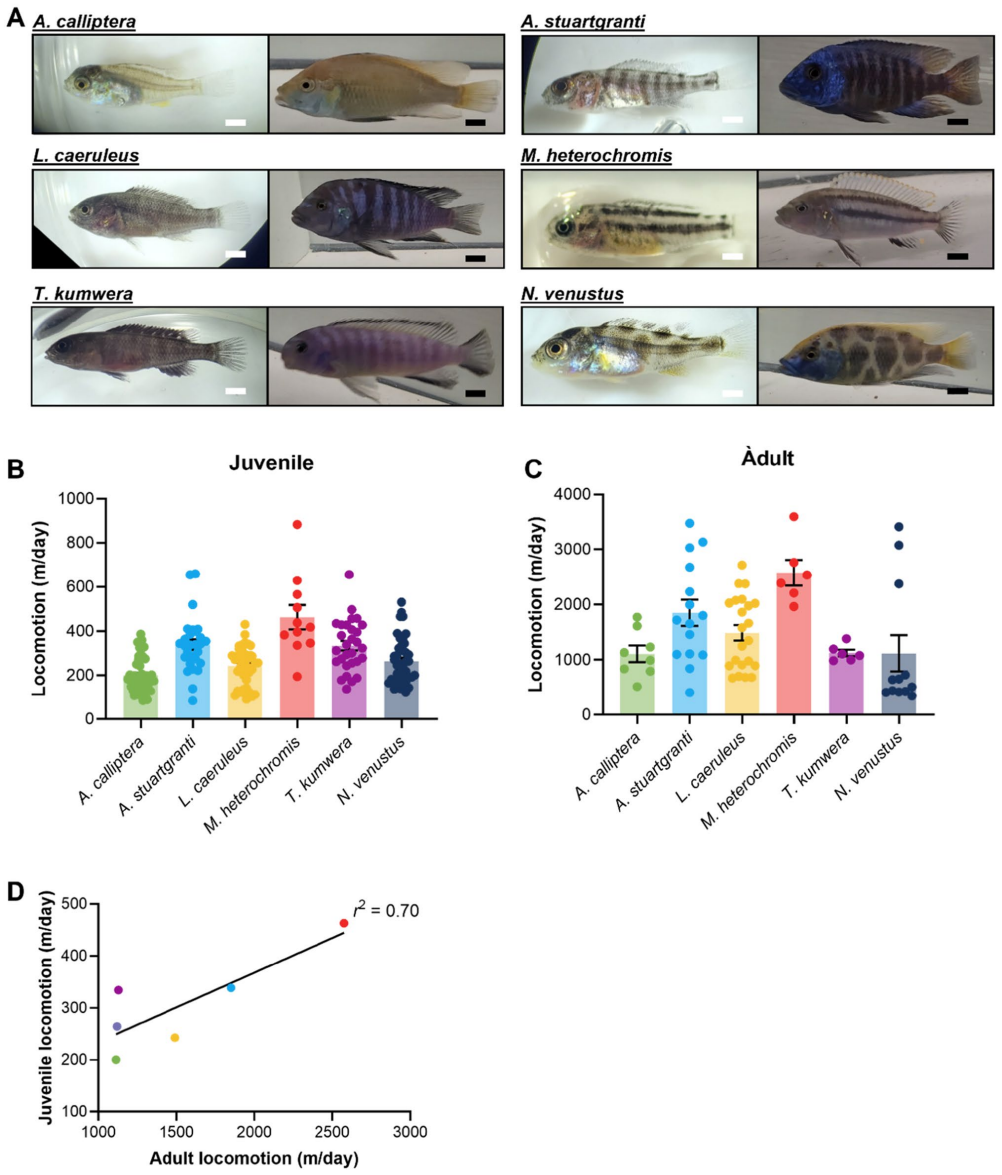
Variation in cichlid activity levels is conserved throughout development. **A** Images of early juvenile (left) and adult (right) cichlid species used in the present study. Juveniles were tested and photographed between 3 and 4 weeks post-fertilization, shortly after the depletion of the yolk sac and at the earliest free-swimming stage. Adults were tested and photographed at full maturity, after the development of nuptial colors. White Scale Bar (Juveniles) = 1 mm. Black Scale Bar (Adults) = 1 cm. **B** Total locomotion of juvenile cichlids over a 24 h period. There is significant variation in total locomotion in juvenile cichlids (ANOVA: *F*_5, 190_ = 15.99, *p* < 0.0001). **C** Total locomotion of adult cichlids over a 24 h period. There is significant variation in total locomotion in adult cichlids (ANOVA: *F*_5, 67_ = 6.42, *p* < 0.0001). **D** Correlation between 24-h locomotion in juveniles and adults. There is a significant correlation between juvenile and adult locomotion (*r*^2^ = 0.70, *p* = 0.0388). Error bars represent ± standard error of the mean

We examined six Lake Malawi cichlids from three distinct lineages (Fig. 1A). The *mbuna* lineage is phylogenetically monophyletic, with species generally inhabiting the near-shore rocky habitat. Three *mbuna* species were utilized here (summarized in Fig. S1Konings 2003; Ribbink et al. 1983): *Labidochromis caeruleus* (Lundo Island), *Melanochromis heterochromis* (Mumbo Island), and *Tropheops kumwera* (Kanchedza Island). All three are territorial and sexually dimorphic as adults. *Labidochromis* species are generally omnivorous, consuming both benthic invertebrates, algae, and plankton. *M. heterochromis* has a similarly omnivorous diet. *T. kumwera* feeds primarily on benthic algae (Ribbink et al. 1983; Konings 2003; Malinsky et al. 2018). *Astatotilapia calliptera* is sister to the *mbuna*, and has been called the “most generalized species in the lake” (Konings 2003). Its generalist designation refers to both diet and habitat, as is one of few Lake Malawi species that inhabits the surrounding river systems. Both *Aulonocara stuartgranti* and *Nimbochromis venustus* are non-*mbuna* species, which is a polyphyletic group of cichlids that generally occupy deeper, open-water, and/or sandy habitats. *A. stuartgranti* feeds on benthic invertebrate that it locates using an enlarged lateral line system, whereas *N. venustus* is an open-water piscivore. All fish were obtained from the aquarium trade. While generation from the wild cannot be verified for these animals, breeding populations are maintained for species, and often to specific locations/populations in the lake, with new individuals introduced to the breeding pool to maintain genetic health.

To measure activity in cichlids, we used systems similar to those established in other fish species including zebrafish and *A. mexicanus* (Yoshizawa et al. 2015; Jaggard et al. 2018; Yokogawa et al. 2007). For juveniles, we used 6-well tissue culture plates, whereas adults were filmed in 2.5-gallon glass aquaria. Sex has not been determined at the age juveniles were tested. For adults, we tested both males and females, in approximately equal numbers, and found no significant differences between males and females in activity level (Fig. S2). Following 24 h of acclimation, individually housed fish were filmed for a 24 h period under standard 14 h light:10 h dark conditions. We noted significant variation in total activity with and between species of juvenile and adults (Fig. 1B, C). For example, *A. calliptera* was the least active in both juveniles and adults, while *M. heterochromis* had the highest activity levels at both stages, suggesting that total activity is conserved across developmental stages (Fig. 1B, C). To directly test this notion, we examined the relationship between total activity in juveniles and adults. A regression revealed a strong positive relationship (*r*^2^ = 0.70, *p* = 0.038) across species between juvenile adult locomotor activity (Fig. 1D). While this correlation is based on species averages, rather than individuals (due to the prohibitive difficulty of tracking an individual’s identity over its lifespan), it suggests that activity level as a trait is stable across the lifespan in these fish. Together, these data reveal significant inter-species variation that remains constant across the life cycle.

### Juvenile cichlids exhibit diurnal or nocturnal activity that is largely absent in adults

**Fig. 2:**
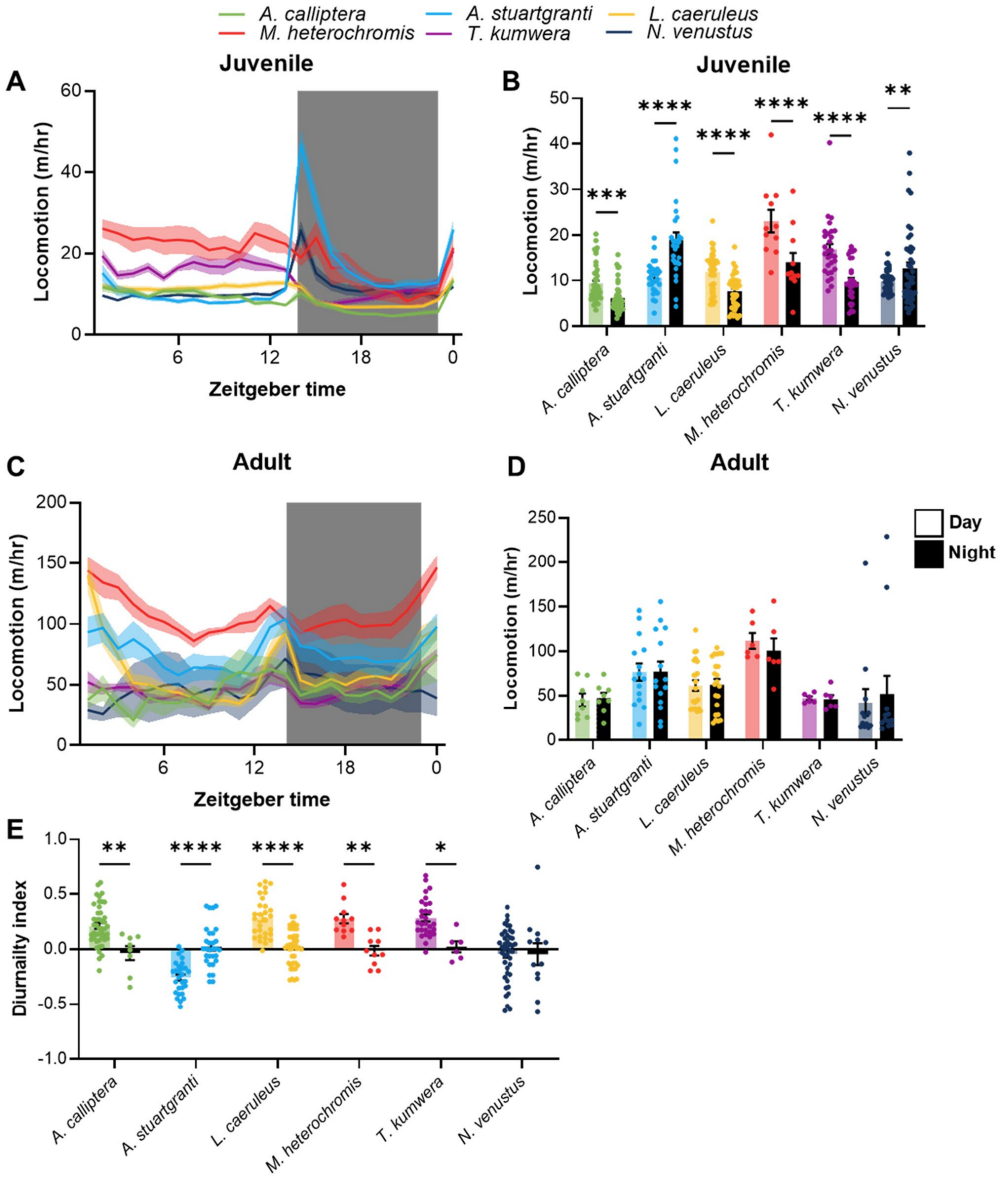
Ontogeny of behavioral rhythms in locomotion. **A** Locomotion profiles of juvenile cichlids over 24 h, binned into 1 h bins, with no smoothing. Shaded area indicates dark period. **B** Average hourly locomotion of juvenile cichlids during the day and night. There was a significant effect of time on locomotion (two-way repeated-measures ANOVA: *F*_1, 190_ = 22.35, *p* < 0.0001). In addition, we found a significant effect of species (*F*_5, 190_ = 16.40, *p* < 0.0001), and a significant interaction between species and time of day (*F*_5, 190_ = 36.54, *p* < 0.0001). **C** Locomotion profiles of adult cichlids over 24 h, binned into 1 h bins, with no smoothing. Shaded area indicates dark period. **D** Average hourly locomotion of juvenile cichlids during the day and night. There was a significant effect of species (two-way repeated-measures ANOVA: *F*_5, 63_ = 3.888, *p* = 0.0039), but not time of day, on locomotion. **E** Strength of behavioral rhythms in juvenile and adult cichlids. 1 indicates total diurnality; -1 indicates total nocturnality. There was a significant effect of developmental stage on diurnality (two-way ANOVA: *F*_1,280_ = 21.92, *p* < 0.0001). We also found a significant effect of species (*F*_5, 280_ = 16.65, *p* < 0.0001), and a significant interaction (*F*_5, 280_ = 16.82, *p* < 0.0001)

We next sought to understand whether rest-activity patterns changed between life-history stages. In juveniles, we identified significant day–night differences in activity patterns across all six species tested (Fig. 2A, B). In total, there were four diurnal species (*A. calliptera*, *L. caeruleus*, *M. heterochromis*, and *T. kumwera*) and two nocturnal species (*A. stuartgranti* and *N. venustus*). Conversely, there were no significant differences between day and nighttime activity across all six species when tested during adulthood (Fig. 2C, D). These results were confirmed when we computed a diurnality index, which compares daytime to nighttime activity in individual fish (Fig. 2E) (Lloyd et al. 2021). By calculating the amount of daytime activity per hour, to the amount of nighttime activity, we can quantifiably define the degree of diurnality or nocturnality in individual fish (See methods for additional description). In juveniles, there was significantly more time-of-day activity preference in five of the six species tested compared to adults of the same species (Fig. 2E). Thus, activity differences across the circadian cycle appear to be more significant in early juvenile cichlids compared to adult fish under individually housed conditions.

It is possible that that the daily activity patterns are transient, and that a single-day recording does not capture their complexity. To verify our findings, we repeated experiments in juvenile and adult *L. caeruleus*, recording activity over a 3 day period. In both cases, the activity patterns were consistent with single-day recordings; juvenile fish were diurnal, while adults lacked rhythms (Fig. S3). Therefore, the activity patterns and ontogenic differences are maintained over the 3 day protocol.

**Fig. 3:**
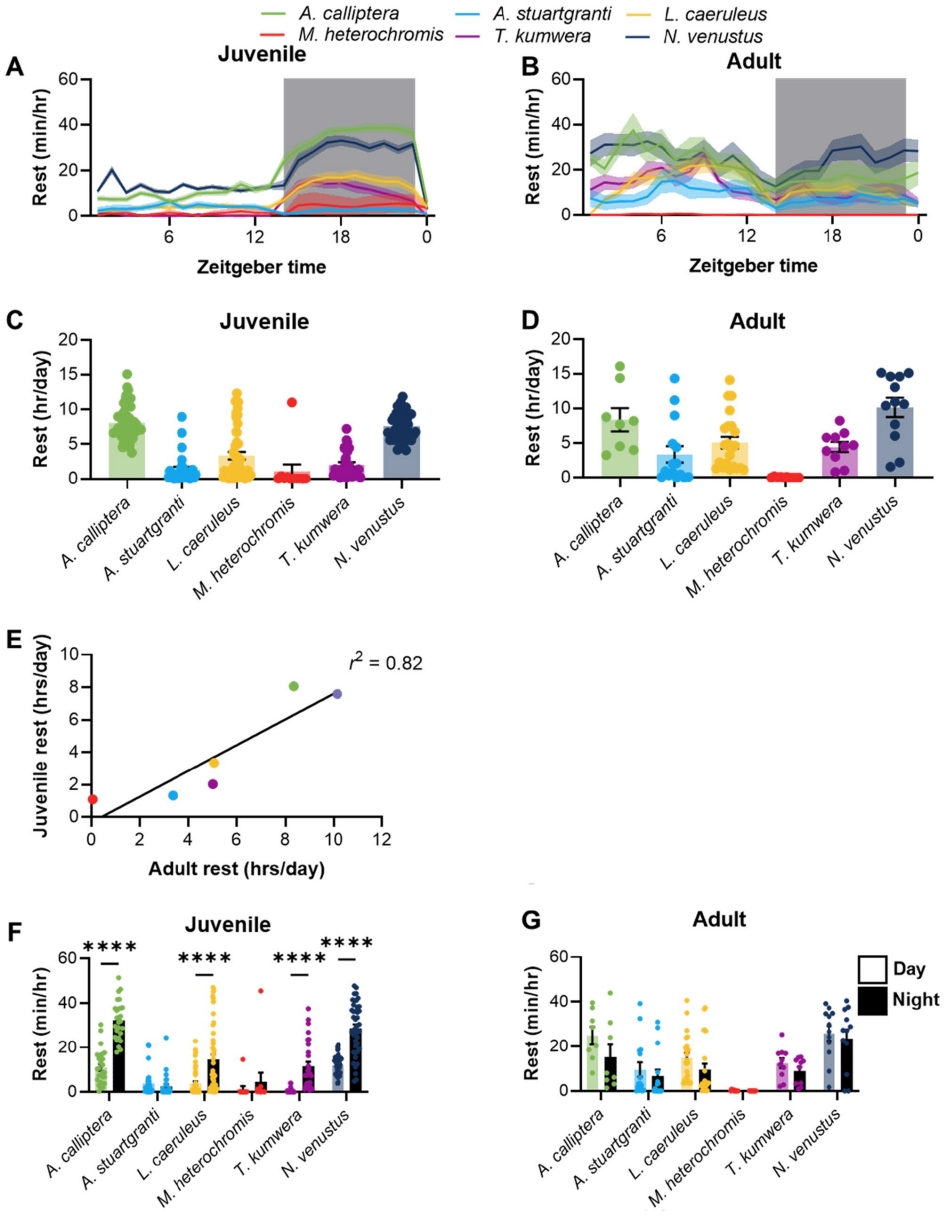
Ontogeny of behavioral rhythms in rest. **A** Rest profiles in juvenile cichlids over 24 h, in 1 h bins, with no smoothing. Shaded area indicates dark period. **B** Rest profiles in adult cichlids over 24 h, in 1 h bins, with no smoothing. Shaded area indicates dark period. **C** Total rest amounts over 24 h in juvenile cichlids. There is significant variation in total rest amount in juvenile cichlids (ANOVA: *F*_5, 190_ = 42.4, *p* < 0.0001). **D** Total rest amounts over 24 h in adult cichlids. There is significant variation in total rest amount in adult cichlids (ANOVA: *F*_5, 67_ = 8.792, *p* < 0.0001). **E** Correlation between juvenile and adult rest amounts. There is significant correlation between juvenile and adult rest (*r*^2^ = 0.82, *p* < 0.0128). **F** Average hourly rest amount of juvenile cichlids during the day and night. There was a significant effect of time on rest (two-way repeated-measures ANOVA: *F*_1, 179_ = 138.5, *p* < 0.0001). We also found a significant effect of species (*F*_5, 179_ = 36.55, *p* < 0.0001), and a significant interaction (*F*_5, 179_ = 15.94, *p* < 0.0001). **G** Average hourly rest amount of adult cichlids. There was a significant effect of time (two-way ANOVA: *F*_1, 142_ = 4.628, *p* = 0.033), but post hoc analysis revealed no significant differences. We also found a significant effect of species on rest amount (*F*_5, 142_ = 13.92, *p* < 0.0001). Error bars represent ± standard error of the mean

Across taxa, the timing of behavioral quiescence or rest is modulated by the circadian clock and linked to daily activity patterns (Bhadra et al. 2017; Foulkes et al. 2016). In many cases, periods of rest are consolidated into defined periods across the circadian cycle in a manner that is not observable by simply examining total activity. While this is often studied as sleep, the behavioral criteria associated with sleep have not been identified in cichlids, and are challenging to define across species. It is possible, however, to quantify periods of inactivity or rest. To examine whether the timing of rest differs across life stages, we therefore compared rest periods of 1 min or longer, a timeframe of inactivity that is used to define sleep in related species (Jaggard et al. 2019). There were significant differences in total rest across juvenile and adult species (Fig. 3A–D). Similar to the analysis of activity, there was consistency in the duration of rest between juveniles and adults. For example, *M. heterochromis* had the lowest levels of rest in juveniles and adults, whereas *A. calliptera* and *N. venustus* had the highest level at botdevelopmental stages. Indeed, a regression analysis revealed a strong relationship (*r*^2^ = 0.82, *p* = 0.012) between juvenile and adult rest duration (Fig. 3E). Therefore, the duration of total rest is maintained throughout development across multiple species of cichlids.

We next compared the amount of rest over the light and dark periods. Juvenile *A. calliptera*, *L. caeruleus,* and *T. kumwera* displayed robust patterns in rest regulation with increased nighttime rest, while the fourth diurnal species *M. heterochromis* trended in the same direction (Fig. 3F), suggesting that diurnality is associated with periods of nighttime rest in juvenile cichlids. Conversely, there were no differences in the timing of rest for *A. stuartgranti,* suggesting that nocturnality in this species is driven by swimming velocity during wakefulness, rather than the timing of rest (Fig. 3F). Finally, in *N. venustus,* which is nocturnal at the juvenile stage, there is consolidation of rest during the night (Fig. 3F). For all species tested, there was little difference between daytime and nighttime rest in adults (Fig. 3G). This trait was also stable over multiple days of testing, in both juveniles (Fig. S4A, B), and adults (Fig. S4C, D). We did note significant differences around the transition to day, and night. This crepuscular behavior is potentially meaningful, because it reveals activity during defined time-frames that may not be captured when examining the average day and nighttime activity. Furthermore, crepuscular behavior is thought to be a critical output of the circadian clock that is under evolutionary selection (Okano et al. 2019; Grima et al. 2004) To understand this trait, we calculated a crepuscularity index, which measures the ratio of activity in the hour following the light transitions relative to the rest of the day (see methods for additional detail). We saw notable variation across species in this measurement, with *N. venustus* and *A. stuartgranti* displaying the highest degree of crepuscularity, increasing activity levels by up to twofold in the hours following light transitions; other species showed little-to-no crepuscular tendencies (Fig. S5A). Similar to day-night behavioral rhythms, crepuscular behavior was largely absent in individually housed adult fish (Fig. S5B).

There were no day–night differences in rest across all six populations of adult cichlids, again confirming that the timing of rest activity is more robust in juveniles than adult fish (Fig. 3G). Together, these findings support the notion that rest-activity bouts are more readily consolidated in juvenile fish and reveal separate mechanisms for the emergence of diurnal, nocturnal, and crepuscular activity patterns.

### Social housing can restore diurnal activity in adults

**Fig. 4:**
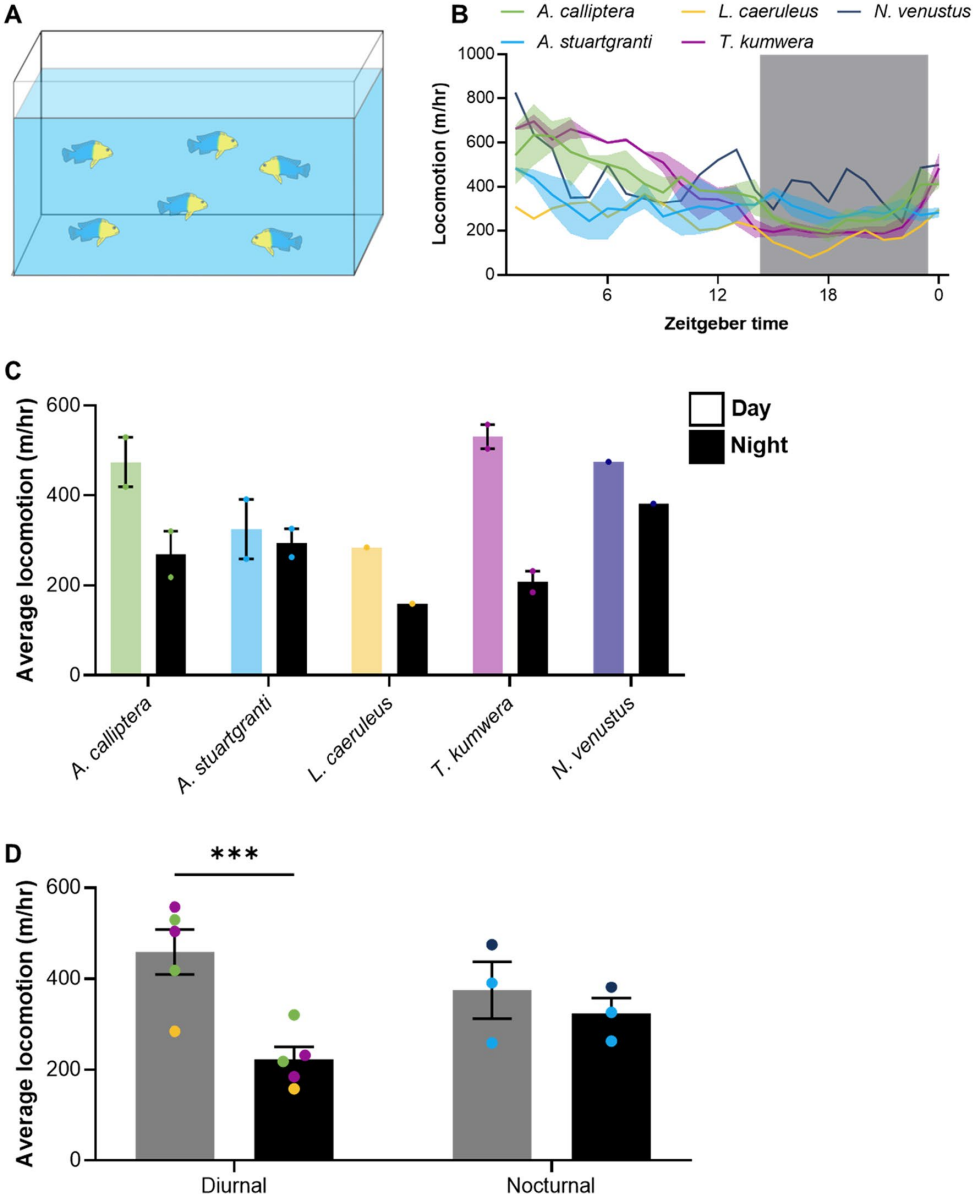
Social context restores rhythms in diurnal cichlids. **A** Schematic of test setup. Adult cichlids are transferred to a ten-gallon testing aquarium, acclimated overnight, and then recorded for 24 h. **B** Average locomotion of cichlid groups over 24 h, in 1 h bins, with no smoothing. Shaded area indicates dark period. **C** Average hourly locomotion of group-housed cichlids, during the day and night. **D** Average hourly locomotion of group-housed cichlids during the day and night, grouped into diurnal and nocturnal species. There was a significant effect of time on locomotion in group-housed cichlids (two-way repeated-measures ANOVA: *F*_1,6_ = 27.25 *p* = 0.002). We also found a significant interaction between time and type (*F*_1, 6_ = 11.23, *p* < 0.0001). Colors of data points indicate species. Error bars represent ± standard error of the mean

Many fish species, including cichlids, are highly social, with animals displaying behavioral differences under solitary and group housing (Tunbak et al. 2020; Patch et al. 2022; Gallego-Abenza et al. 2022; Guo et al. 2022; Solomon-Lane and Hofmann 2019). Except for a small number of studies, nearly all analysis of rest-activity patterns in fishes have used individually housed animals, including cichlids. Social interactions are weaker in juvenile fish, compared to adults, raising the possibility that the lack of rest-activity patterns in adults is an artifact of solitary housing conditions. To examine the effects of social housing on rest-activity patterns, we measure activity patterns in group-housed cichlids comprised of two adult males and four adult females (Fig. 4A). All fish were transferred into testing tanks and given 24 h to acclimate and establish stable social interactions. We tested four different cichlid species under group-housed conditions for 24 h. While we were unable to follow the same individual over the 24 h period due to switching of fish in the automated tracking system, we were able to apply automated analysis to measure group activity. Three of these species tested (*A. calliptera*, *L. caeruleus*, and *T. kumwera*) trended toward recovery of diurnal behavior (Fig. 4C). There was little difference in the species *A. stuartgranti* or *N. venustus,* which are nocturnal as juveniles. To examine the broader trends of sleep in group-housed fish, we combined analysis of nocturnal and diurnal species. Social housing induced robust diurnality in species that are diurnal as juveniles, while there was no differences between day and night activities in the two nocturnal species (Fig. 4D). To verify results in longer term recordings, we repeated the analysis with *L. caeruleus* over a 3 day recording. Across two trials, groups trended toward diurnality across in each trial, confirming results obtained during single-day recordings (Fig. S6). These findings suggest diurnal juveniles’ species likely maintain activity patterns in adulthood, but the fish only display these behaviors under group-housed conditions.

**Fig. 5:**
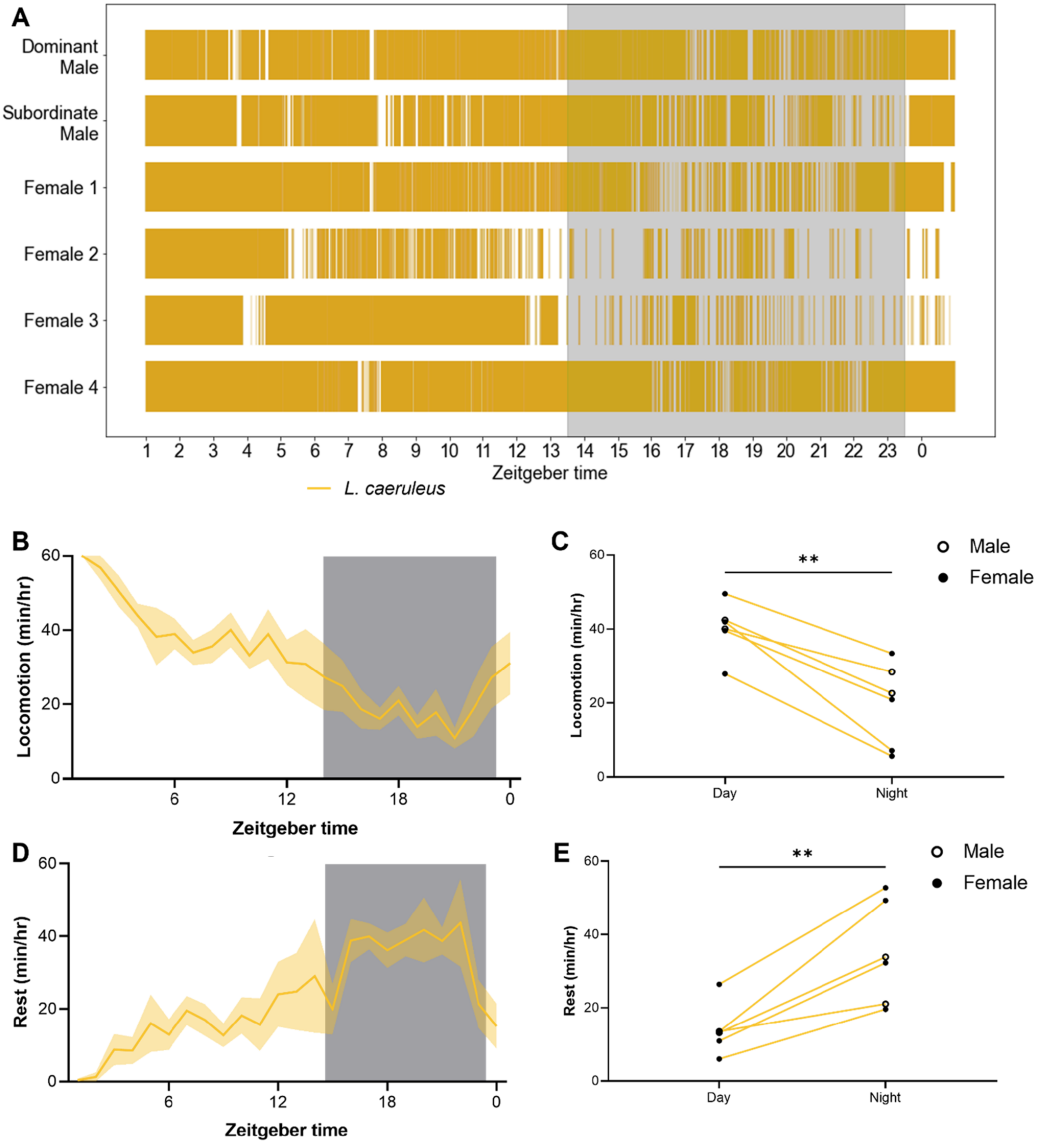
Individual variation in behavioral rhythms in a social context. **A** Ethogram of active swimming behavior of *L. caeruleus* over 24 h, in a social context. Colored regions indicate periods of active swimming. Shaded area indicates dark period. **B** Profile of locomotion in group-housed *L. caeruleus*, quantified as minutes of active swimming per hour. Shaded area indicates dark period. **C** Average hourly locomotor activity of group-housed *L. caeruleus* during the day and night. Group-housed *L. caeruleus* are significantly more active during the day (paired *t* test: *t*_5_ = 6.413, *p* = 0.0014). **D** Rest profile of group-housed *L. caeruleus* over 24 h. Shaded area indicates dark period. **E** Average hourly rest of group-housed *L. caeruleus* during the day and night. Group-housed *L. caeruleus* consolidate rest during the dark period (paired *t* test: *t*_5_ = 5.198, *p* = 0.0035). Error bars represent ± standard error of the mean

To examine the behavior of individual fish in more detail, we manually annotated behavior for a single species over the full 24 h testing period. We chose to examine *L. caeruleus* because of their robust diurnal behavior as group-housed adults and individually housed juveniles. Fish behavior was manually analyzed in Behavioral Observation Research Interactive Software (BORIS) for active swimming over the 24 h cycle (Friard and Gamba 2016). We developed an ethogram across all six fish with rest-activity timing (Fig. 5A). We observed robust diurnal activity across all male and female individuals (Fig. 5B, C). Further, the duration of rest bouts was greater during the night period (Fig. 5D, E). These findings confirm that diurnality is not sex-specific, and generalizable across the dominance structure.

## Discussion

### The importance of context in behavioral studies

The genetic, morphological, and behavioral diversity of African cichlids provides an exceptional model for understanding trait evolution (Salzburger 2018). Despite a myriad comparative studies in this system, very few have focused on rest or activity patterns. Here, we characterize the behavior of six species of Lake Malawi cichlids at multiple developmental stages, highlighting a role for ontogeny and social context in the regulation of activity. Previously, we characterized rest-activity patterns in 11 species of Lake Malawi cichlids at the late juvenile, or sub-adult stage, identifying seven species without activity preferences, as well as two diurnal species and one nocturnal species (Lloyd et al. 2021). The lack of activity preferences across the majority of species was surprising given the ubiquity of circadian rhythms and the robust diurnal or nocturnal behaviors across many species of animals that have been studied to date. Here, we examined species that also seemed to lack robust activity patterns as adults, but showed that these behaviors are present when animals were examined at an early juvenile stage or within a social context. Of note, *T. kumwera* showed diurnal activity patterns in our previous study, when tested at the sub-adult stage. Here, we found that this behavioral rhythm is present from the earliest developmental timepoints, but requires social interactions by the onset of sexual maturity. However, placing the adult fish in a social context restored diurnal rhythmicity, underscoring the importance of a social context for maintenance of behavioral rhythms in this species. These findings highlight the complexity of animal behaviors, and suggest that many of the species previously examined may possess innate activity rhythms when examined in a different context.

The role of circadian and sleep regulators across development remains poorly understood. For example, in humans, sleep varies significantly across the life cycle, but little is known about the factors that regulate pediatric sleep (Frank 2020). In fruit flies, genes regulating sleep and activity in larvae differ from those regulating sleep in adult flies, suggesting that the genetic basis of the consolidation of activity patterns is specific to developmental stages (Brown et al. 2019; Szuperak et al. 2018). Here, we find that total locomotor activity and duration of rest appear to be robust to life-history stage, whereas the timing of rest-activity patterns is dependent upon ontogeny, raising the possibility that there are genetic and contextual differences regulating the timing, but not the amount of activity. The cichlid species examined here reach sexual maturity at ~ 6 months. This stage is associated by an increase in sex-specific hormones that is associated with aggression, territoriality, mating behaviors, and dominance hierarchies (Maruska et al. 2022). The gain of these behaviors seems to be associated with the loss of activity pattern robustness, suggesting that natural social conditions are required for the expression of diurnal or nocturnal rhythms. Although sexual maturity appeared to coincide with the loss of activity patterns in isolation, we found no significant differences in activity level between males and females of any species tested, in either the individually or group tested fish. Whether the lack of rhythms in individually housed adults is due to the absence of an essential environmental cue, or represents a secondary stress response remains unclear, but there are accounts of social isolation in cichlids leading to increased stress, aggression, and reduced cognitive performance (Earley et al. 2006; Galhardo and Oliveira 2014; Brandão et al. 2015). The loss of behavioral activity may be a related phenomenon. Taken together, our data suggest that understanding the rest-activity patterns in adults will require further testing in diverse ethological contexts.

It is notable that total rest activity appears to be robust to life-history stage, which suggests that it is more hard-wired compared to rest-activity patterns. If true, this would suggest greater potential to modulate activity rhythms as populations face new environmental and/or social contexts. While the correlations in overall activity and rest were based on species averages, rather than individuals (due to the prohibitive difficulty of tracking an individual’s identity over long periods of development), it suggests that activity level as a trait is stable across the lifespan in these fish. Differences in the variance of this trait across species (e.g., very high variance in *N. venustus*, low variance in *T. kumwera*) suggest that this is complex trait that is regulated by many factors. In addition, as we have shown that adult activity is modulated by other factors such as group housing, it is notable that we identified a relatively strong correlation based on this single factor. Our findings suggest the evolution of highly disparate locomotor patterns, diurnality and nocturnality, are influenced by environmental/social context. These findings raise the possibility that the evolution of complex social behaviors co-evolves with rest-activity patterns to contribute to the regulation of circadian behavior.

### Biological mechanisms of circadian regulation

The hypothalamus is the primary regulator of circadian behavior and outputs from the suprachiasmatic nucleus are thought to convey diurnal or nocturnal behavior (Steindal and Whitmore 2020; Moore and Whitmore 2014). In zebrafish, the SCN contains core-transcriptional clock genes common in mammals suggesting a conserved function in the regulation of locomotor patterns, although the presence of many light-responsive tissues in teleosts suggests clock regulation may be more complicated than in mammals (Steindal and Whitmore 2020). In *Drosophila* through mammals, social interactions contribute to the regulation of sleep and circadian behavior. In mammals, social interactions impact clock entrainment. For example, social defeat decreases the amplitude of the core-clock gene Per2 in the periphery, as well as sleep homeostasis (Radwan et al. 2021; Ota et al. 2020). There is reduced complexity of social interactions in young juvenile fish that displayed robust rhythms (Solomon-Lane and Hofmann 2019; Hesse and Thünken 2014). However, we find that activity patterns are absent in individually housed adult fish. These findings raise the possibility that unlike mammals, stress that impairs circadian function comes from solitary housing, rather than social stress. Indeed, in *Drosophila*, solitary housing is associated with sleep dysregulation and disruption of circadian gene expression (Li et al. 2021; Wang et al. 2022). Understanding both the SCN outputs, and how they are modulated by social context provides a mechanism for understanding how context-dependent regulation of activity patterns evolved. Our findings raise the possibility that these outputs are modulated by brain circuits involved in regulation of social behavior, or other context-specific regulators of behavior (O’Connell and Hofmann 2011; Chen and Hong 2018). Regulation of social behavior is a complex process, involving the modulation of complex decision-making by integration of sensory inputs, internal hormonal states, and prior knowledge of social interactions. One possibility is that hormonal changes caused by social isolation impair the function of the circadian clock, leading to impaired regulation of circadian rhythms.

### Adaptive significance

Development of diversity in behavioral rhythms in cichlids may be a type of habitat partitioning, with cichlid species altering the timing of their behaviors to take advantage of reduced competition for resources, such as food and territory (Lloyd et al. 2021). The presence of larger nocturnal predators in Lake Malawi, such as the Cornish Jack *Mormyrops anguilloides*, presents a constraint to this adaptation, requiring nocturnal cichlids to develop strategies for avoiding predation at night (Arnegard and Carlson 2005). It has been previously reported that *A. stuartgranti* possesses widened lateral line canals which enable them to detect prey in the dark, but this same adaptation may also enable detection and evasion of predators during the dark period (Schwalbe et al. 2012). It is interesting that both nocturnal species are also crepuscular, and both are predatory. There is evidence that predator–prey interactions are highest during the twilight hours, and crepuscularity may be an adaptation in predators to facilitate access to both day-active and night-active prey (Campanella et al. 2019; Helfman 1986). Also notable is that neither species recovered rhythmicity when placed in a group context. This may reflect differences in the level of social interaction in these fish in the wild, such that it is not group context, but rather a yet unidentified factor that is required for adult activity rhythms. In short, there is still much to learn about the regulation of activity patterns in this system, and testing hypotheses about the adaptive significance of these behaviors will require the application of phylogenetic methods on a much larger sampling of taxa.

## Challenges and future directions

It has been found that changes in not just light quantity, but also composition (color), occur during twilight, and that the circadian clock machinery is responsive to this change (Oosterhout et al. 2012; Walmsley et al. 2015_)._. Because our study used constant levels of white light throughout the day, we were unable to evaluate the effects of changes in light quality on behavioral rhythms; future studies may address this question using simulated dusk/dawn transitions.

The duration of circadian experiments provide a significant challenge for analyzing data across multiple species. Here, we used multiple approaches to quantify locomotor activity in group-housed fish. First, we used Ethovision to track total activity. While this system is capable of tracking individual fish in a shared arena, the tracks regularly cross-over preventing measurements of activity in individuals over a 24 h period. To identify the activity of individuals, we visually quantified behavior using BORIS. While this approach allows for reliable behavioral measurements in individual fish it lacks precise quantification. Given the time-consuming nature of the analysis, we only analyzed a single experiment of six fish. Recent development in automated tracking, including DeepLabCut and IDTracker, may provide applications for long-term automated tracking (Mathis et al. 2018; Romero-Ferrero et al. 2019). These systems have been effectively applied to measure social behaviors and activity in populations of animals (Mathis et al. 2018; Wiltschko et al. 2020). Further, we have tracked large groups of *A. mexicanus* to examine social interactions (Patch et al. 2022). While these applications have yet to be applied to long-term analysis of rest-activity patterns, the rapid improvements in processing speed and accuracy are likely to allow for analysis of sleep and circadian regulation of activity under social contexts.

It is widely recognized that rest-activity patterns are regulated by many life-history and environmental traits (Arble et al. 2015; Patke et al. 2020). While this study examines the effects of social context on circadian behavior in five species, these represent a small fraction of > 500 species in Lake Malawai alone. Given the complexity of social behaviors in cichleds and the diversity of ecological nichs, a better of the relationship between sleep and social context will require the study of a broader number of species in laboratory and wild settings.

The growing use of animal models in rest-activity regulation has provided unprecedented insight into the genetic, neural, and evolutionary processes that govern these behaviors. However, the vast majority of studies still test animals under individually housed conditions, because it provides a simpler method of data acquisition and the removal of variables that may impact behavior. The results of this study suggest that developmental stage and environmental conditions can have profound effects on behavioral regulation, and highlight the need for a more thorough investigation of social influences and other factors that may play a role in the regulation of behavior.

## Materials and methods

### Fish husbandry

Cichlids used for experiments were reared following standard protocols (Patch et al. 2022; Gallego-Abenza et al. 2022) approved by the Texas A&M University Institutional Animal Care and Use Committee. Cichlids were housed in the Keene fish facilities at Texas A&M University at a water temperature of 28.5 °C, on a 14 h:10 h light:dark cycle. Adult cichlids were fed TetraMin tropical flakes (TetraMin) twice a day. Juvenile cichlids were fed live *Artemia* brine shrimp twice daily.

When not being used for experiments, all cichlids were group housed in tanks consisting only of conspecifics. While we could not guarantee a constant sex ratio across all species while not being tested, home tanks always contained multiple individuals of both sexes.

### Fish breeding

Breeding was facilitated by the inclusion of clay pots in the cichlid’s home tanks, which provided arenas for mating behaviors. Breeding occurred spontaneously, and females were periodically visually inspected for an enlarged buccal cavity, evidence of fertilized eggs. Mouthbrooding females were allowed to carry their embryos until the hatching stage (approximately 3–5 days). A waiting period prior to extraction increased survival rates of the offspring in our hands.

To extract fertilized eggs, the mouthbrooding female was transferred to a holding tank and briefly restrained by hand, while the mouth was gently opened with the pad of the thumb. The buccal cavity was gently stroked to facilitate egg removal. Following extraction, the female was returned to her home tank, and the eggs were transferred to a 1000 mL capacity Erlenmeyer flask (VWR, 10545-842) with an air stone bubbler to maintain oxygen levels. Eggs were left to develop until free-swimming, and the yolk mostly depleted (approximately 3–4 weeks), at which point they were transferred to the experimental setup immediately. Cichlid staging followed previous work, whereby the onset of juvenile stage is coincident with yolk absorption and exogenous foraging (Fujimura and Okada 2007).

### Behavior measurements

For experiments testing locomotor activity in juvenile fish, juveniles were transferred to individual wells of 6-well culture plates (Falcon, 353046), and placed on a light box constructed of white 1/8″ high-density polyethylene plastic (TAP Plastics), which allowed for even diffusion of light to facilitate automated tracking. Light boxes were lit from below with infrared light, using 850 nm LED strips (Environmental Lights). Fish were acclimated to the testing chambers overnight; behavioral recording began the following day at ZT1, and ran for 24 h. Juveniles were filmed from above, at 15 frames per second with a USB camera (LifeCam Studio 1080p, Microsoft), modified to remove the IR-blocking filter, and with an IR-pass filter (Edmund optics, 43-948) added to ensure consistent lighting during the light and dark periods.

For experiments testing locomotor activity in isolated adult fish, adults were transferred to 2.5 gallon tanks (Carolina Biological Supply, 671226), fitted on the floor and walls with custom-cut white corrugated plastic (3 mm thick), allowing for even diffusion of light to facilitate automated tracking. Tanks were lit from behind with infrared light. Fish were acclimated to the testing chambers overnight; behavioral recording began the following day at ZT1, and ran for 24 h. Adults were filmed from the side, with the same recording equipment described above.

For experiments testing locomotor activity in group-housed adult fish, adults were transferred to ten-gallon tanks (Carolina Biological Supply, 671230), fitted on the floor and walls with custom-cut white corrugated plastic, and lit from behind with infrared light. Fish were acclimated to the testing chambers overnight; behavioral recording began the following day at ZT1, and ran for 24 h. Each group consisted of 2 male and 3–4 female conspecifics.

### Automated behavioral tracking

Acquired videos were processed in Ethovision XT 15 (Noldus), and positional data over the 24 h period were extracted and analyzed using a custom-made Python script (Supplementary Material) to calculate locomotor activity on an hourly basis. Analysis of rest patterns was carried out as previously described, with a threshold of 4 cm/s for adults, and 12 mm/s for juveniles (Lloyd et al. 2021). Bouts of inactivity greater than 60 s were considered rest. These numbers were determined empirically, based on visual observation of the animal’s behavior, to distinguish passive drift from active swimming. Although a range of values provided similar results, these were chosen to maintain consistency with our previous work in cichlids and other fish species.

For group-housed experiments, locomotor activity was calculated using Ethovision’s Social Interaction Module (Noldus). Due to limitations of the software, the identities of individual fish could not be maintained over the 24 h period, so the average locomotion within each group was used for subsequent analysis.

### Manual behavior scoring

To assess individual behavioral rhythms within group-housed cichlids, a single 24-h video was manually scored in BORIS, an interactive behavior logging software (Friard and Gamba 2016). Active swimming behavior was logged as a state event (on/off), according to the following criteria: “Focal fish can be seen actively swimming with appendicular movements even though the pace may change throughout the duration of one bout of movement”. A full 24 h was logged for each fish (*N* = 2 males, *N* = 4 females) in a single video. The identity of the dominant male was determined by his larger size and nuptial coloration pattern. The females were also identified by size, with the largest female designated “Female 1” and the smallest “Female 4”. A single scorer performed all manual activity logging, to avoid inter-individual differences in the evaluation of criteria. Data for each fish were exported in the “Aggregated Events” format, and then analyzed using a custom-made Python script (Supplementary Material) to extract hourly measurements of locomotion. Rest bouts were calculated from the aggregated movement data, with any period of inactivity greater than 60 s considered a rest bout.

### Statistical analysis

ANOVAs were performed to identify differences in activity or rest between species. To identify differences between activity or rest in the light versus dark, a two-way repeated-measures ANOVA was carried out. To identify differences in degree of diurnality, a two-way ANOVA was performed. Šidák’s multiple comparisons post hoc test was used for all post hoc comparisons. All statistical testing was carried out using InStat software (GraphPad Prism 9.5).

Diurnality was calculated as described previously, as an “activity change ratio”, with ${A}_{R}= \frac{D-N}{D+N}$, where *D* and *N* are average hourly activity during the day and night. Crepuscularity was calculated as ${C}_{R}= \frac{C}{NC}-1$, where *C* is equal to the average activity in the hours following light transitions (i.e., dawn and dusk), and *NC* is equal to the average activity across the rest of the day. In this formulation, 0 represents no change in activity following a light transition, and 1 represents a 100% increase in activity levels (Lloyd et al. 2021).

### Supplementary Information

Below is the link to the electronic supplementary material.

Supplementary file1 (DOCX 2489 KB)

## Data Availability

Data is fully available upon request.

## References

[CR1] Arble DMDM, Bass J, Behn CDCD, Butler MPMP, Challet E, Czeisler C, Depner CM, Elmquist J, Franken P, Grandner MA (2015). Impact of sleep and circadian disruption on energy balance and diabetes : a summary of workshop discussions. Sleep.

[CR2] Arnegard ME, Carlson BA (2005). Electric organ discharge patterns during group hunting by a mormyrid fish. Proc Biol Sci.

[CR3] Bass J, Lazar MA (2016). Circadian time signatures of fitness and disease. Science.

[CR4] Beale A, Guibal C, Tamai TK, Klotz L, Cowen S, Peyric E, Reynoso VH, Yamamoto Y, Whitmore D (2013). Circadian rhythms in Mexican blind cavefish *Astyanax mexicanus* in the lab and in the field. Nat Commun.

[CR5] Bhadra U, Thakkar N, Das P, Pal Bhadra M (2017). Evolution of circadian rhythms: from bacteria to human. Sleep Med.

[CR6] Brandão ML, Braithwaite VA, Gonçalves-de-Freitas E (2015). Isolation impairs cognition in a social fish. Appl Anim Behav Sci.

[CR7] Brown EB, Slocumb ME, Szuperak M, Kerbs A, Gibbs AG, Kayser MS, Keene AC (2019). Starvation resistance is associated with developmentally specified changes in sleep, feeding and metabolic rate. J Exp Biol.

[CR8] Campanella F, Auster PJ, Taylor JC, Muñoz RC (2019). Dynamics of predator-prey habitat use and behavioral interactions over diel periods at sub-tropical reefs. PLoS ONE.

[CR9] Cavallari N, Frigato E, Vallone D, Fröhlich N, Lopez-Olmeda JF, Foà A, Berti R, Sánchez-Vázquez FJ, Bertolucci C, Foulkes NS (2011). A blind circadian clock in cavefish reveals that opsins mediate peripheral clock photoreception. PLoS Biol.

[CR10] Ceinos RM, Frigato E, Pagano C, Fröhlich N, Negrini P, Cavallari N, Vallone D, Fuselli S, Bertolucci C, Foulkes NS (2018). Mutations in blind cavefish target the light-regulated circadian clock gene, period 2. Sci Rep.

[CR11] Chen P, Hong W (2018). Neural circuit mechanisms of social behavior. Neuron.

[CR12] Duboué ER, Keene AC, Borowsky RL (2011). Evolutionary convergence on sleep loss in cavefish populations. Curr Biol.

[CR13] Earley R, Edwards J, Aseem O, Felton K, Blumer L, Karom M, Grober M (2006). Social interactions tune aggression and stress responsiveness in a territorial cichlid fish (*Archocentrus nigrofasciatus*). Physiol Behav.

[CR14] Foulkes NS, Whitmore D, Vallone D, Bertolucci C (2016). Studying the evolution of the vertebrate circadian clock: the power of fish as comparative models. Adv Genet.

[CR15] Frank MG (2020). The ontogenesis of mammalian sleep: form and function. Curr Sleep Med Rep.

[CR16] Friard O, Gamba M (2016). BORIS: a free, versatile open-source event-logging software for video/audio coding and live observations. Methods Ecol Evol.

[CR17] Froese R, Pauley D. www.fishbase.org. Fishbase

[CR18] Fryer G, Iles TD (1972). The cichlid fishes of the great lakes of Africa. Their biology and evolution.

[CR19] Fujimura K, Okada N (2007). Development of the embryo, larva and early juvenile of Nile tilapia *Oreochromis niloticus* (Pisces: Cichlidae). Developmental staging system. Dev Growth Differ.

[CR20] Galhardo L, Oliveira RF (2014). The effects of social isolation on steroid hormone levels are modulated by previous social status and context in a cichlid fish. Horm Behav.

[CR21] Gallego-Abenza M, Boucherie PH, Bugnyar T (2022). Early social environment affects attention to social cues in juvenile common ravens *Corvus corax*. R Soc Open Sci.

[CR22] Grima B, Chélot E, Xia R, Rouyer F (2004). Morning and evening peaks of activity rely on different clock neurons of the Drosophila brain. Nature.

[CR23] Guo H, Näslund J, Thomassen ST, Larsen MH (2022). Social isolation affects intra-specific interaction behaviour and reduces the size of the cerebellar brain region in juvenile Atlantic salmon *Salmo salar*. J Fish Biol.

[CR24] Helfman GS (1986). Fish behaviour by day, night and twilight. The behaviour of teleost fishes.

[CR25] Herrero MJ, Pascual M, Madrid JA, Sánchez-Vázquez FJ (2005). Demand-feeding rhythms and feeding-entrainment of locomotor activity rhythms in tench (*Tinca tinca*). Physiol Behav.

[CR26] Hesse S, Thünken T (2014). Growth and social behavior in a cichlid fish are affected by social rearing environment and kinship. Naturwissenschaften.

[CR27] Hurd MW, Debruyne J, Straume M, Cahill GM (1998). Circadian rhythms of locomotor activity in zebrafish. Physiol Behav.

[CR28] Jaggard JB, Stahl BA, Lloyd E, Prober DA, Duboue ER, Keene AC (2018). Hypocretin underlies the evolution of sleep loss in the Mexican cavefish. Elife.

[CR29] Jaggard JB, Lloyd E, Lopatto A, Duboue ER, Keene AC (2019). Automated measurements of sleep and locomotor activity in Mexican cavefish. J vis Exp.

[CR30] Joiner WJ (2016). Unraveling the evolutionary determinants of sleep. Curr Biol.

[CR31] Keene AC, Duboue ER (2018). The origins and evolution of sleep. J Exp Biol.

[CR32] Kocher TD (2004). Adaptive evolution and explosive speciation: the cichlid fish model. Nat Rev Genet.

[CR34] Konings A (2003). Malawi cichlids in their natural habitat.

[CR35] Kornfield I, Smith PF (2000). African cichlid fishes: model systems for evolutionary biology. Annu Rev Ecol Syst.

[CR36] Kronfeld-Schor N, Visser ME, Salis L, van Gils JA (2017). Chronobiology of interspecific interactions in a changing world. Philos Trans R Soc Lond B Biol Sci.

[CR37] Li W, Wang Z, Syed S, Lyu C, Lincoln S, O’Neil J, Nguyen AD, Feng I, Young MW (2021). Chronic social isolation signals starvation and reduces sleep in Drosophila. Nature.

[CR38] Lloyd E, Chhouk B, Conith AJ, Keene AC, Albertson RC (2021). Diversity in rest-activity patterns among Lake Malawi cichlid fishes suggests a novel axis of habitat partitioning. J Exp Biol.

[CR39] Mack KL, Jaggard JB, Persons JL, Roback EY, Passow CN, Stanhope BA, Ferrufino E, Tsuchiya D, Smith SE, Slaughter BD (2021). Repeated evolution of circadian clock dysregulation in cavefish populations. PLoS Genet.

[CR40] Malinsky M, Svardal H, Tyers AM, Miska EA, Genner MJ, Turner GF, Durbin R (2018). Whole-genome sequences of Malawi cichlids reveal multiple radiations interconnected by gene flow. Nat Ecol Evol.

[CR41] Maruska KP, Anselmo CM, King T, Mobley RB, Ray EJ, Wayne R (2022). Endocrine and neuroendocrine regulation of social status in cichlid fishes. Horm Behav.

[CR42] Mathis A, Mamidanna P, Cury KM, Abe T, Murthy VN, Mathis MW, Bethge M (2018). DeepLabCut: markerless pose estimation of user-defined body parts with deep learning. Nat Neurosci.

[CR43] Mattis J, Sehgal A (2016). Circadian rhythms, sleep, and disorders of aging. Trends Endocrinol Metab.

[CR44] Mohawk JA, Green CB, Takahashi JS (2012). Central and peripheral circadian clocks in mammals. Annu Rev Neurosci.

[CR45] Moore HA, Whitmore D (2014). Circadian rhythmicity and light sensitivity of the zebrafish brain. PLoS ONE.

[CR46] O’Connell LA, Hofmann HA (2011). The vertebrate mesolimbic reward system and social behavior network: a comparative synthesis. J Comp Neurol.

[CR47] Okano K, Kaczmarzyk JR, Dave N, Gabrieli JDE, Grossman JC (2019). Sleep quality, duration, and consistency are associated with better academic performance in college students. NPJ Sci Learn.

[CR48] Ota SM, Hut RA, Riede SJ, Crosby P, Suchecki D, Meerlo P (2020). Social stress and glucocorticoids alter PERIOD2 rhythmicity in the liver, but not in the suprachiasmatic nucleus. Horm Behav.

[CR49] Patch A, Paz A, Holt KJ, Duboué ER, Keene AC, Kowalko JE, Fily Y (2022). Kinematic analysis of social interactions deconstructs the evolved loss of schooling behavior in cavefish. PLoS ONE.

[CR50] Patke A, Young MW, Axelrod S (2020). Molecular mechanisms and physiological importance of circadian rhythms. Nat Rev Mol Cell Biol.

[CR51] Radwan B, Yanez Touzet A, Hammami S, Chaudhury D (2021). Prolonged exposure to social stress impairs homeostatic sleep regulation. Front Neurosci.

[CR52] Ribbink AJ, Marsh BA, Marsh AC, Ribbink AC, Sharp BJ (1983). A preliminary survey of the cichlid fishes of rocky habitats in Lake Malawi. South Afr J Zool.

[CR53] Romero-Ferrero F, Bergomi MG, Hinz RC, Heras FJH, de Polavieja GG (2019). idtracker.ai: tracking all individuals in small or large collectives of unmarked animals. Nat Methods.

[CR54] Salzburger W (2018). Understanding explosive diversification through cichlid fish genomics. Nat Rev Genet.

[CR55] Sánchez-Vázquez FJ, Madrid JA, Zamora S, Iigo M, Tabata M (1996). Demand feeding and locomotor circadian rhythms in the goldfish, *Carassius auratus*: dual and independent phasing. Physiol Behav.

[CR56] Schwalbe MAB, Bassett DK, Webb JF (2012). Feeding in the dark: lateral-line-mediated prey detection in the peacock cichlid *Aulonocara stuartgranti*. J Exp Biol.

[CR57] Solomon-Lane TK, Hofmann HA (2019). Early-life social environment alters juvenile behavior and neuroendocrine function in a highly social cichlid fish. Horm Behav.

[CR58] Steindal IAF, Whitmore D (2020). Zebrafish circadian clock entrainment and the importance of broad spectral light sensitivity. Front Physiol.

[CR59] Szuperak M, Churgin MA, Borja AJ, Raizen DM, Fang-Yen C, Kayser MS (2018). A sleep state in Drosophila larvae required for neural stem cell proliferation. Elife.

[CR60] Tunbak H, Vazquez-Prada M, Ryan TM, Kampff AR, Dreosti E (2020). Whole-brain mapping of socially isolated zebrafish reveals that lonely fish are not loners. Elife.

[CR61] van Oosterhout F, Fisher SP, van Diepen HC, Watson TS, Houben T, VanderLeest HT, Thompson S, Peirson SN, Foster RG, Meijer JH (2012). Ultraviolet light provides a major input to non-image-forming light detection in mice. Curr Biol.

[CR62] Walmsley L, Hanna L, Mouland J, Martial F, West A, Smedley AR, Bechtold DA, Webb AR, Lucas RJ, Brown TM (2015). Colour as a signal for entraining the mammalian circadian clock. PLoS Biol.

[CR63] Wang Z, Lincoln S, Nguyen AD, Li W, Young MW (2022). Chronic sleep loss disrupts rhythmic gene expression in Drosophila. Front Physiol.

[CR64] Wiltschko AB, Tsukahara T, Zeine A, Anyoha R, Gillis WF, Markowitz JE, Peterson RE, Katon J, Johnson MJ, Datta SR (2020). Revealing the structure of pharmacobehavioral space through motion sequencing. Nat Neurosci.

[CR65] Yokogawa T, Marin W, Faraco J, Pézeron G, Appelbaum L, Zhang J, Rosa F, Mourrain P, Mignot E (2007). Characterization of sleep in zebrafish and insomnia in hypocretin receptor mutants. PLoS Biol.

[CR66] Yoshizawa M, Robinson BG, Duboué ER, Masek P, Jaggard JBJ, O’Quin KEK, Borowsky RLR, Jeffery WRW, Keene ACA, Duboue E (2015). Distinct genetic architecture underlies the emergence of sleep loss and prey-seeking behavior in the Mexican cavefish. BMC Biol.

[CR67] Zhdanova IV, Wang SY, Leclair OU, Danilova NP (2001). Melatonin promotes sleep-like state in zebrafish. Brain Res.

