# Supplementary figures and images for: Ontogeny and social context regulate the circadian activity patterns of Lake Malawi cichlids
Source: J Comp Physiol B. 2023 Nov 1;194(3):299–313. doi: 10.1007/s00360-023-01523-3 (PMC11233325; doi:10.1007/s00360-023-01523-3)

**Supplemental Figures**


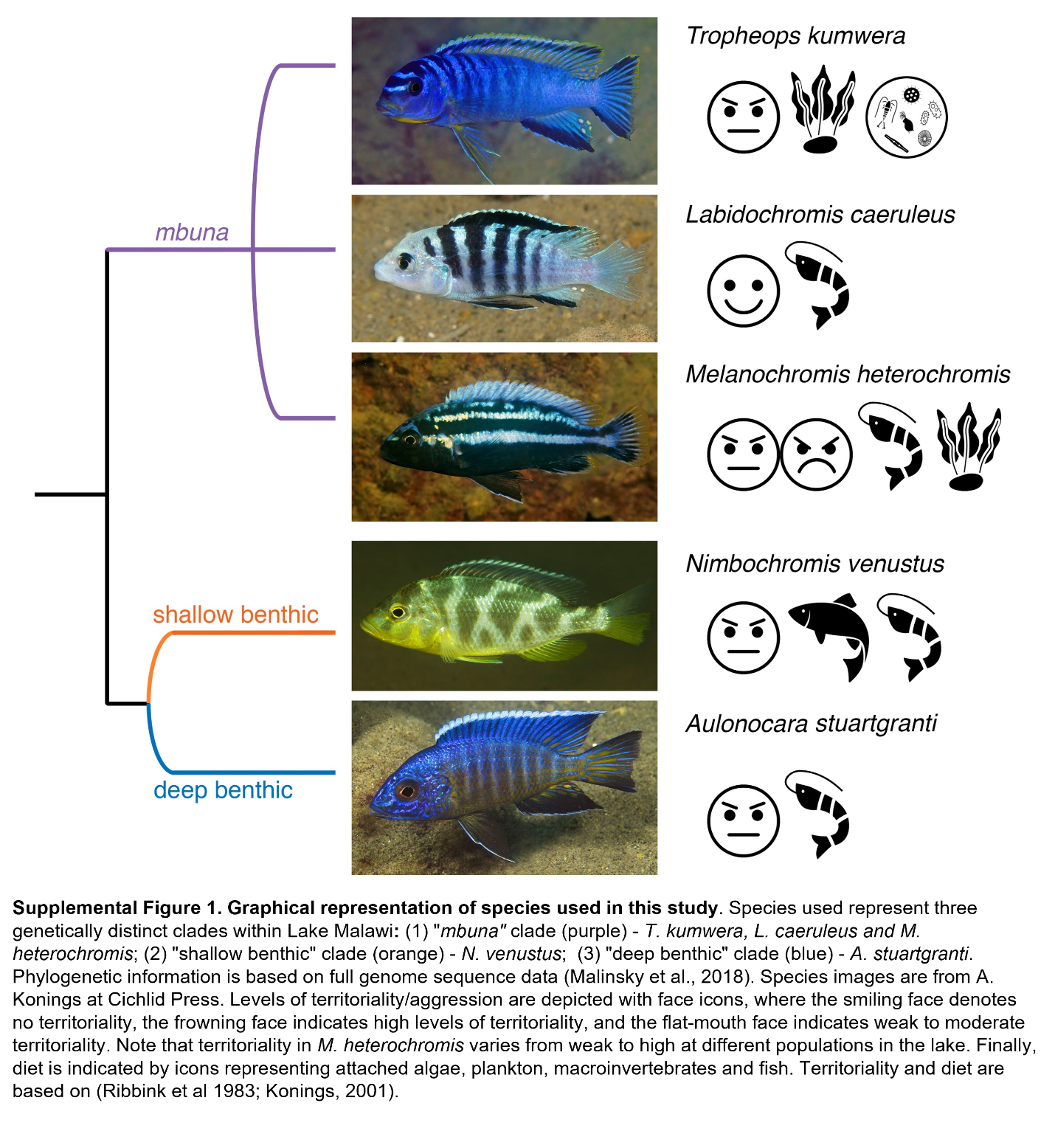


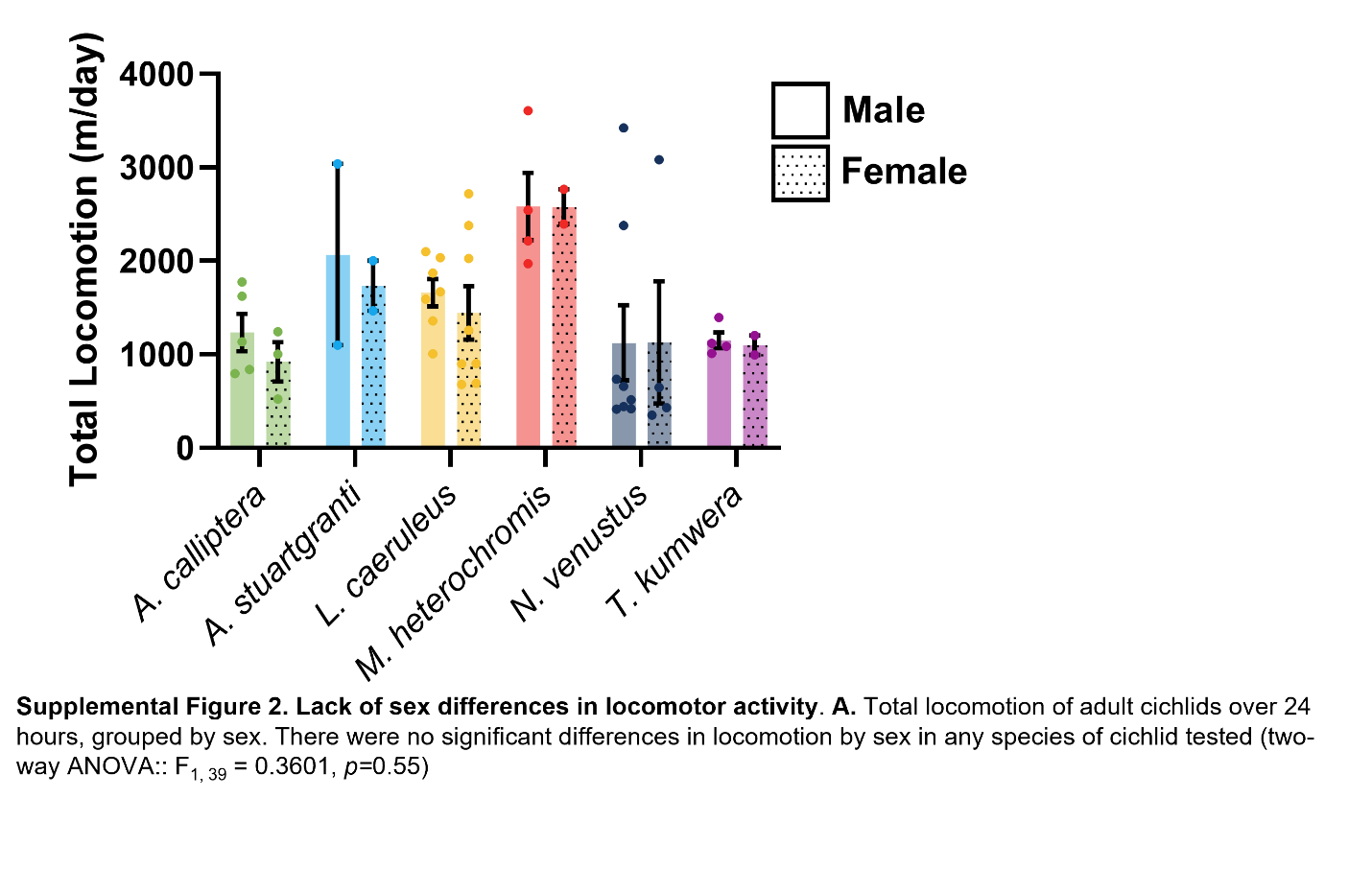


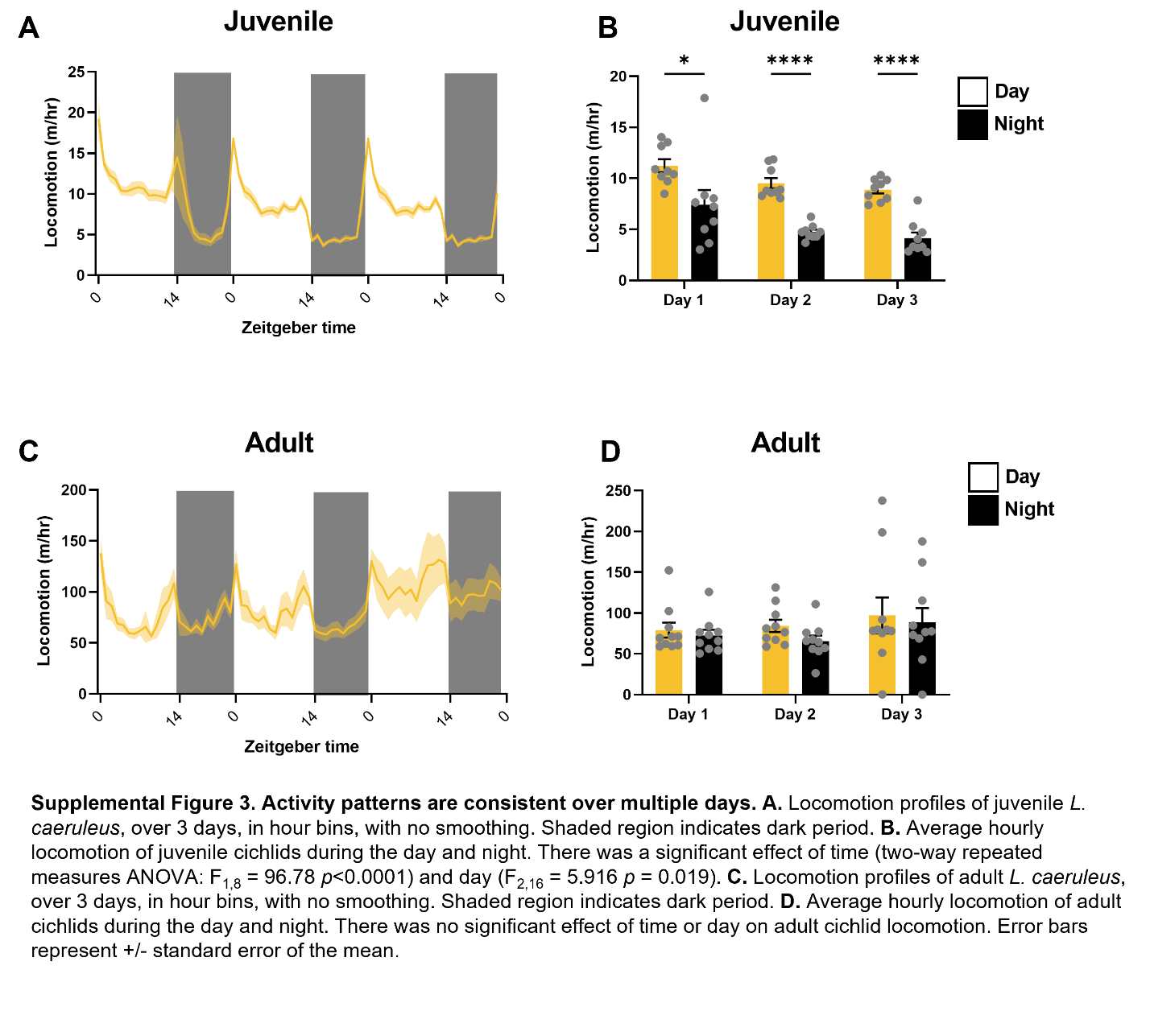


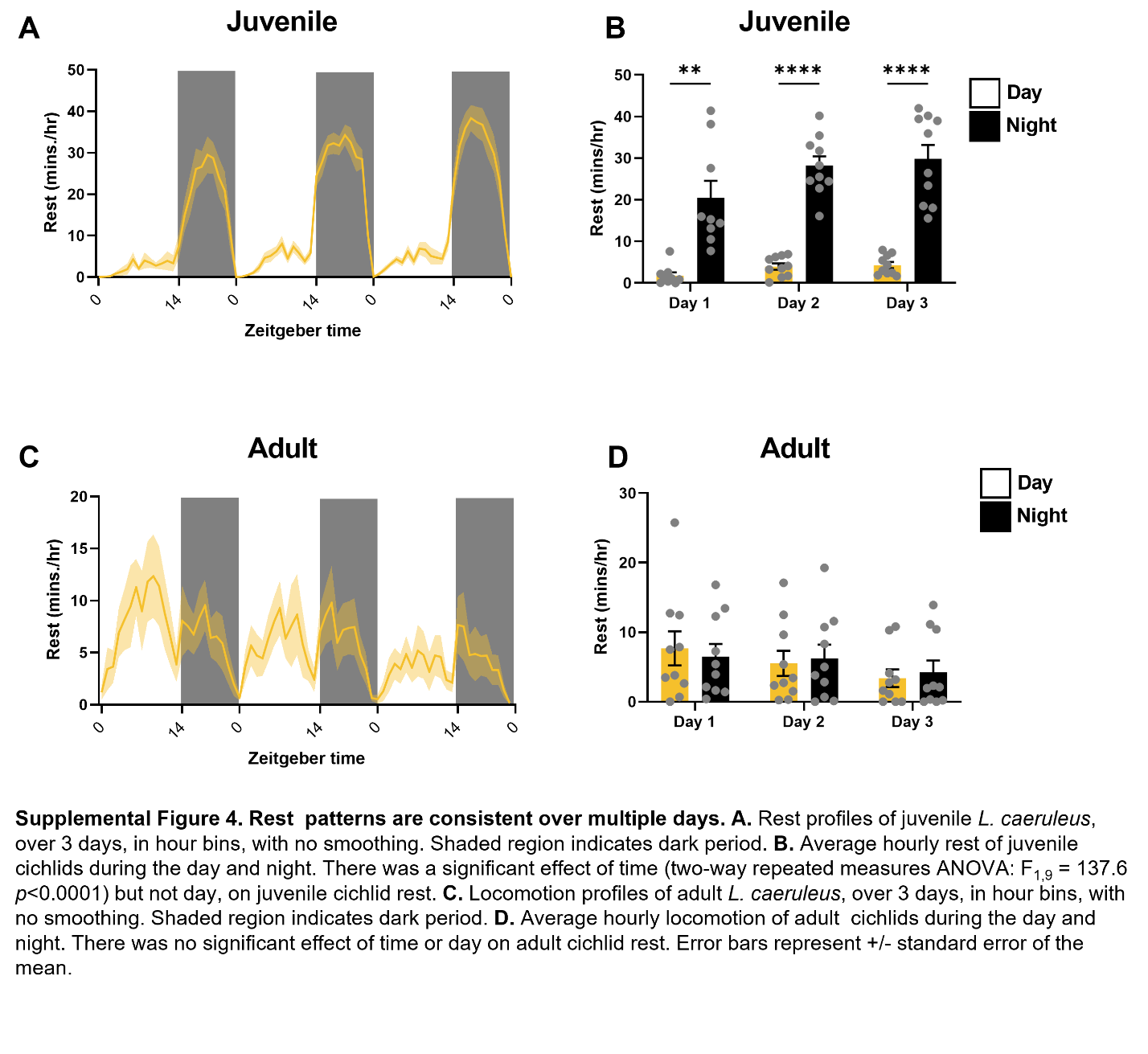

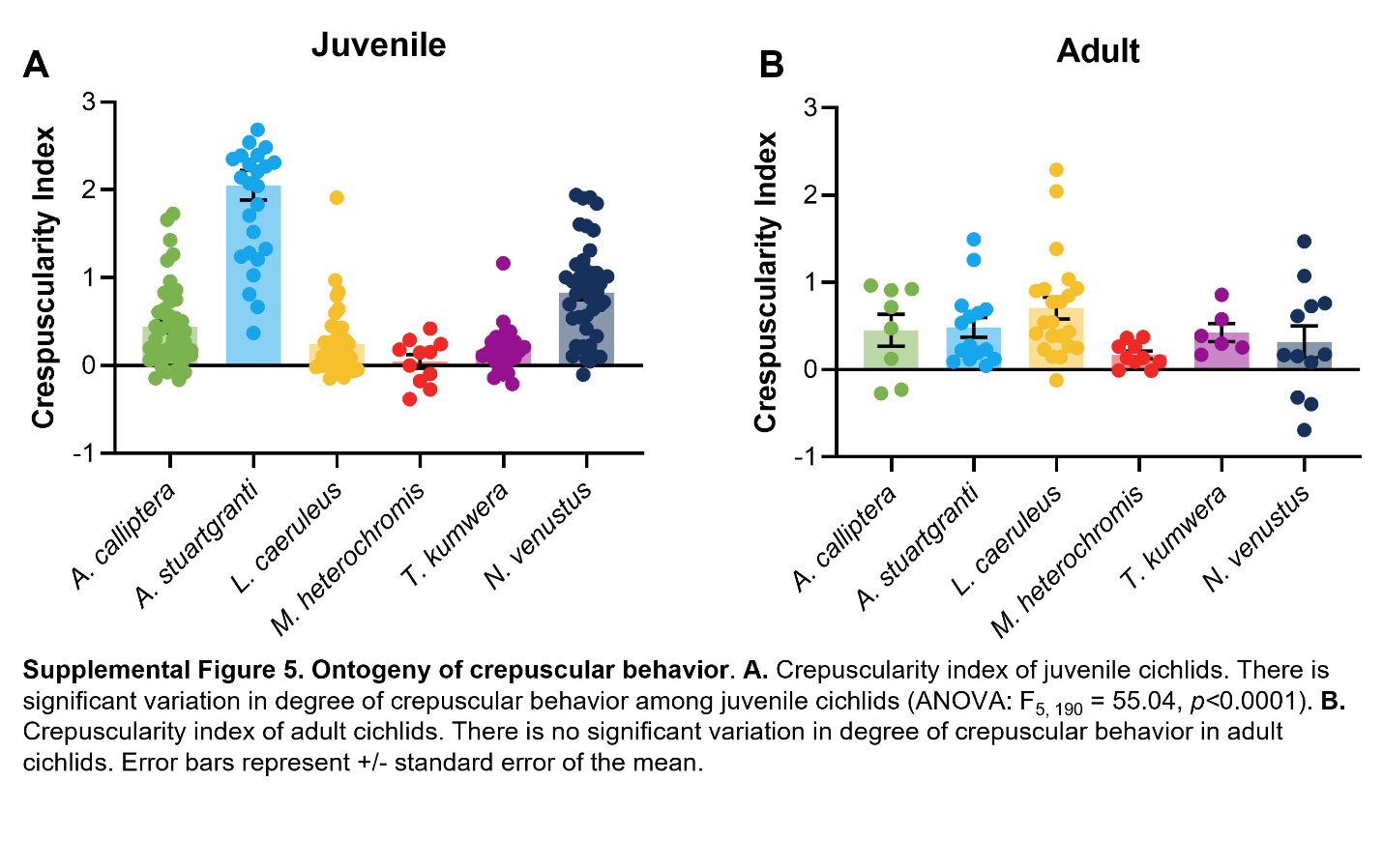


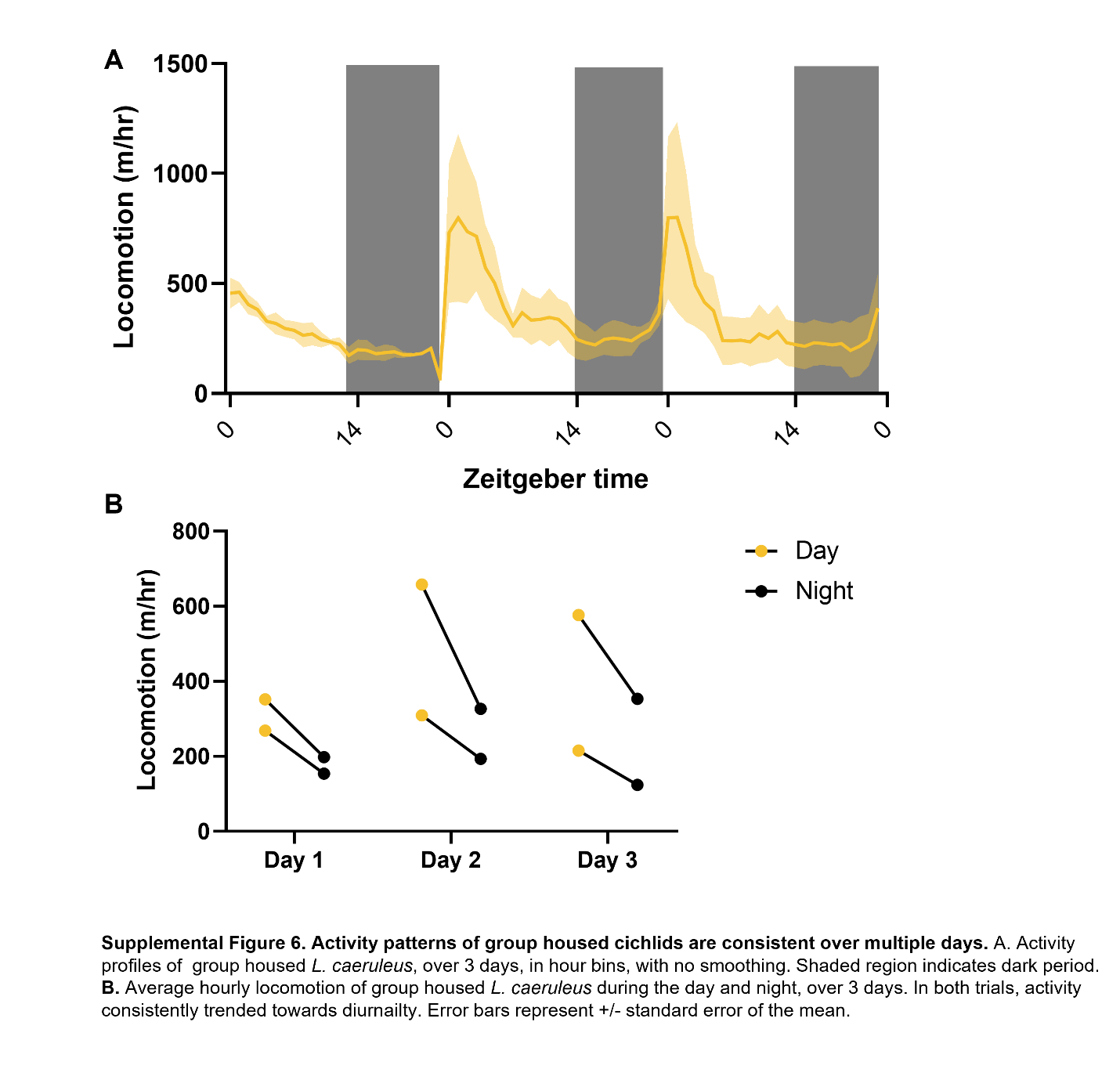

Supplement: Supplementary file 1 — Supplementary file1 (DOCX 2489 KB) [file 360_2023_1523_MOESM1_ESM.docx]
